# MicroRNA-1 (miR-1) inhibits gastric cancer cell proliferation and migration by targeting MET

**DOI:** 10.1007/s13277-015-3358-6

**Published:** 2015-04-01

**Authors:** Chao Han, Yubing Zhou, Qi An, Feng Li, Duolu Li, Xiaojian Zhang, Zujing Yu, Lili Zheng, Zhenfeng Duan, Quancheng Kan

**Affiliations:** 1 Department of Clinical Pharmacology, The First Affiliated Hospital of Zhengzhou University, Zhengzhou, 450052 China; 2 Department of Infectious Diseases, The First Affiliated Hospital of Zhengzhou University, Zhengzhou, China; 3 Department of Endocrinology and Metabolism, The First Affiliated Hospital of Zhengzhou University, Zhengzhou, China; 4 Sarcoma Biology Laboratory, Center for Sarcoma and Connective Tissue Oncology, Massachusetts General Hospital, Boston, MA USA

**Keywords:** Gastric cancer, miR-1, Non-coding RNA, Tumor suppressor gene, Target therapy

## Abstract

MicroRNAs (miRs) are short endogenous non-coding RNAs that act as posttranscriptional regulatory factors of gene expression. Downregulation of miR-1 has been reported in gastric cancer; however, the mechanisms underlying its functions via target genes in gastric cancer remain largely unknown. The purpose of this study was to investigate the mechanism by which miR-1 inhibits gastric cancer cell proliferation and migration. The effects of miR-1 on gastric cancer cell proliferation and migration were determined by MTT and wound-healing assays. Cell protein expression of the miR-1 target gene MET was analyzed by Western blotting. Finally, MET expression was evaluated by immunohistochemistry in a stomach tumor tissue microarray (TMA). Ectopic expression of miR-1 inhibited proliferation and migration in both AGS and SGC-7901 gastric cancer cell lines. miR-1 directly targets the MET gene and downregulates its expression. MET siRNA also inhibited proliferation and migration in both cell lines. Immunohistochemistry revealed significantly higher MET expression levels in gastric cancer tissues compared with matched adjacent non-cancer tissues. These findings indicate that the miR-1/MET pathway is a potential therapeutic target due to its crucial role in gastric cancer cell proliferation and migration.

## Introduction

Gastric cancer is the fourth most common malignancy worldwide. It is more common in men and in developing countries, including East Asian and Eastern European nations. There were approximately 700,000 gastric cancer deaths in 2012, making this malignancy the third leading cause of cancer death after lung and liver cancers [1, 2]. Treatment of stomach cancer may include surgery, adjuvant chemotherapy, and/or radiation therapy. Despite the demonstrated benefits of these treatments, gastric cancer remains virtually incurable with the metastatic disease. Therefore, it is urgent to improve the overall survival rate of patients with gastric cancer by identifying novel therapeutic strategies.

MicroRNAs (miRs) are endogenous small non-coding RNA molecules that bind to complementary sequences in specific regions of multiple target mRNAs to act as posttranscriptional gene expression regulators [3, 4]. MiRs impair gene expression by repressing translation or promoting mRNA degradation. Dysregulation of miRs therefore interferes with many biological processes such as cell proliferation, metabolism, differentiation, apoptosis, immunity, and development [5–9]. Moreover, multiple studies have shown that miRs play an important role in human cancers; they could be used as diagnostic and prognostic cancer biomarkers and even be applied to therapy [10, 11]. MicroRNA-1 (miR-1) is a known tumor suppressor that is downregulated in several types of malignancies such as lung cancer, colorectal cancer, prostate cancer, bladder cancer, rhabdomyosarcoma, and chordoma [12–17]. It has also been shown that miR-1 is underexpressed in gastric cancer compared to normal stomach epithelium, in line with its potential tumor suppressor role [18]. However, other studies have demonstrated that miR-1 levels are increased in gastric cancer samples compared with the control samples [19]. These inconsistencies indicate that miR-1 and its function need further characterization.

The MET gene is a direct target of miR-1 [4, 10]. MET is a known oncogene that encodes a cell surface receptor tyrosine kinase, which is upregulated in a variety of human cancers [20–22]. The activation of MET is due to binding of hepatocyte growth factor (HGF), followed by MET dimerization and auto-phosphorylation. These events contribute to tumor growth, metastasis, migration, and drug resistance [23, 24]. Furthermore, MET has been used as a target to improve cancer therapy and ameliorate the sensitivity of chemotherapy in different cancers [25, 26]. MET has been predicted and confirmed to be a target gene for multiple miRs, including miR-1.

In this study, we investigated the functions of miR-1 in gastric cancer cell proliferation and migration. Specifically, we focused on the miR-1 target gene MET to determine its expression in gastric tumor tissues.

## Materials and methods

### Cell culture

Human gastric cancer cell lines AGS and SGC-7901 were purchased from the American Type Culture Collection (ATCC, Manassas, VA, USA). Both cell lines were grown in RPMI-1640 medium containing 10 % fetal bovine serum (FBS), 100 units/ml of penicillin, and 100 μg/ml streptomycin. Cells were cultured in a humidified atmosphere containing 5 % CO_2_ at 37 °C.

### MicroRNA mimics transfection and siRNA treatment

The following RNA products were used in this research: hsa-miR-1 mimics, negative control miR mimics, small interfering RNA (siRNA), and negative control siRNA mimics; these RNA products were chemically synthesized by Shanghai GenePharma Co. Ltd. A total of 2 × 10^5^ cells in 2 ml of culture medium were seeded per well of a six-well plate 1 day before transfection. For transfection, miR or siRNA sample was mixed with Lipofectamine^TM^ RNAiMax (Invitrogen, CA, USA), then Opti-MEM (Invitrogen, CA, USA) was added dropwise into the well after incubation for 10 min.

### RNA extraction and real-time PCR for quantification of miR-1

For detecting the expression of miR, total RNA from cells were harvested by TRIzol (Invitrogen, CA, USA) reagent. SYBR Green real-time PCR (RT-PCR) was performed to validate expressed miR-1 after transfection of miR-1 mimics. For mature miR-1 detection, cDNA reverse transcription was performed from total RNA samples using specific miR-1 primers from the SYBR Green MicroRNA Assays and reagents from the miR Reverse Transcription Kit (Novland Biopharma, Shanghai, China).

### Cell proliferation and motility assays

Cells were transfected with various amounts of miR-1 mimics and siRNA by reverse transfection according to the manufacturer’s instructions and plated at a density of 4 × 10^3^ cells per well in 96-well plates. After 72 h, cell proliferation was detected by MTT (Sigma-Aldrich, Saint Louis, MO, USA) assay. For each treatment group, triplicate wells were analyzed for cell viability.

To evaluate cell motility, a wound-healing assay was carried out. AGS cells were plated in six-well plates at 2 × 10^5^ cells per well, and wounds were generated using a micropipette tip. Then, cells were rinsed three times with phosphate-buffered saline and fresh culture medium was added. The residual gap widths were evaluated from photomicrographs after 48 and 72 h of wound establishment. The wound-healing assays were repeated three times. The data were then analyzed using Prism 5.0 software and expressed as mean ± SEM.

### Western blot analysis

AGS and SGC-7901 cells were seeded in six-well plates (2 × 10^5^ cells/well) and cultured in RPMI-1640 containing 10 % FBS for 24 h. After transfection for 72 h, the cells were washed with cold phosphate-buffered saline and subjected to lysis with RIPA lysis buffer (50 mM Tris-HCl, 150 mM NaCl, 1 % Triton X-100, 1 % sodium deoxycholate, 0.1 % SDS, and other protease inhibitors) on ice. Afterward, equal amounts of protein lysate (30 μg) and Precision Plus Protein™ Dual Color Standards (Cat#: 161-0374, Bio-Rad, Hercules, CA, USA) were separated by NuPAGE on 4–12 % bis-tris gel (Invitrogen, CA, USA), transferred onto nitrocellulose membranes and blocked in 5 % non-fat milk for 1 h. Immunoblotting was carried out overnight with diluted polyclonal antibodies against MET (1:500; Santa Cruz Biotechnology, USA), survivin (1:1000), and beta-actin (1:3000). Afterward, the membranes were washed three times with TBS-T and incubated with IRDye 680LT Goat anti-rabbit (H + L) (1: 5000) or IRDye 800CW goat anti-mouse (H + L) (1:15,000; Li-COR Biosciences, Lincoln, NE, USA) for 2 h at room temperature. The bound complexes were detected by using the Odyssey Infrared Imaging System (Li-COR, Lincoln, NE, USA), and images were analyzed with the Odyssey Application Software.

### Tissue microarray assay

To assess MET expression in human gastric cancers, a set of gastric cancer tissue microarray (TMA) was purchased from Shanghai Outdo Biotech (OD-CT-DgStm01-014, Shanghai, China). The TMA contained 75 gastric cancer tissues with matched adjacent non-gastric cancer tissues (Table 1). Rabbit-derived anti-human MET antibody (1:200 dilution, Santa Cruz Biotechnology, USA) was used for immunohistochemical (IHC) detection of the MET protein in TMA samples. Endogenous peroxidase was inhibited by incubation with freshly prepared 3 % hydrogen peroxide containing 0.1 % sodium azide. TMA was successively incubated with goat anti-rabbit antibodies and ExtrAvidin-conjugated horseradish peroxidase. Staining was developed with the diaminobenzidine (DAB) substrate, and sections were counterstained with hematoxylin. The proportion of positively stained tumor cells was staged as follows: 0 (no positive membrane staining of MET tumor cells), 1+ (<25 % positive tumor cells), 2+ (25–50 % positive tumor cells), 3+ (50–75 % positive tumor cells), and 4+ (>75 % positive tumor cells).

**Table 1:** Clinical parameters of gastric cancer tissue microarray

Parameters	n(%)
Age	75
≤45	8(10.67)
45-60	14(18.67)
≥60	53(70.66)
Gender	75
Male	50(66.67)
Female	25(33.33)
Prognosis	25
Survival	14(56)
Non survival	11(44)
Pathological grade	75
I	7(9.33)
II	40(53.33)
III	27(36)
IV	1(1.33)

### Statistical analysis

Statistical analysis was performed using the GraphPad Prism 5.0 software. For comparison between MET expression and clinical/pathologic variables, a two-sided Student *t* test was used. Statistical significance is described in the figures and respective legends. For comparison between MET expression and immune infiltrates, a one-way ANOVA analysis was used. *P* < 0.05 was considered statistically significant.

## Results

### Ectopic expression of miR-1 inhibits gastric cancer cell proliferation and motility in vitro

**Fig. 1:**
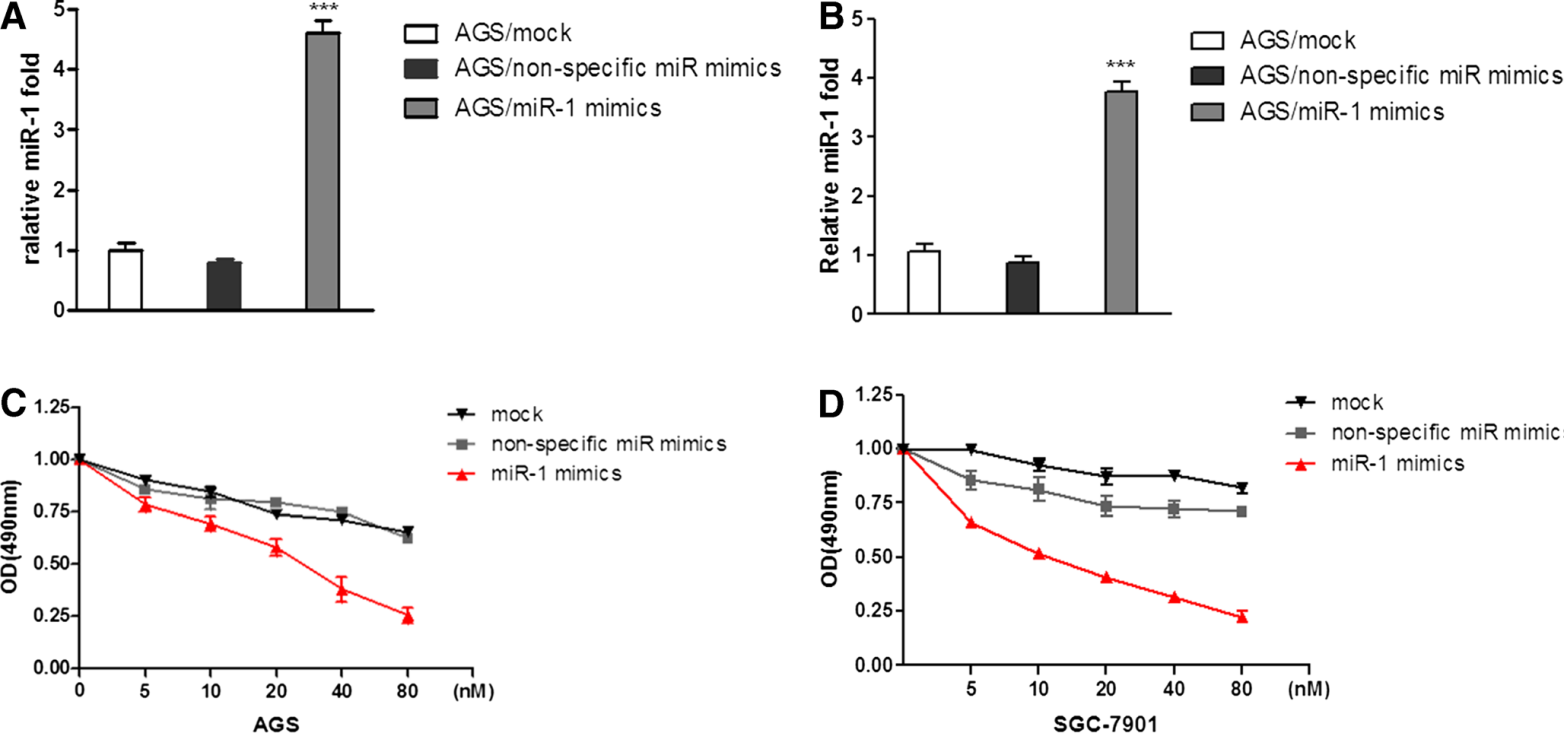
Transfection with miR-1 inhibits gastric cancer AGS and SGC-7901 cell growth. **a**, **b** To verify the expression levels of miR-1 in these two gastric cancer cell lines after transfecting miR-1 mimics for 48 h. **c**, **d** AGS and SGC-7901 cells were transfected with miR-1 mimics and non-specific miR mimics at 5–80 nM, respectively, and incubated for up to 72 h in a medium containing 10 % FBS. Cell growth was measured by MTT-based cell proliferation assay. The experiment was performed in triplicate

**Fig. 2:**
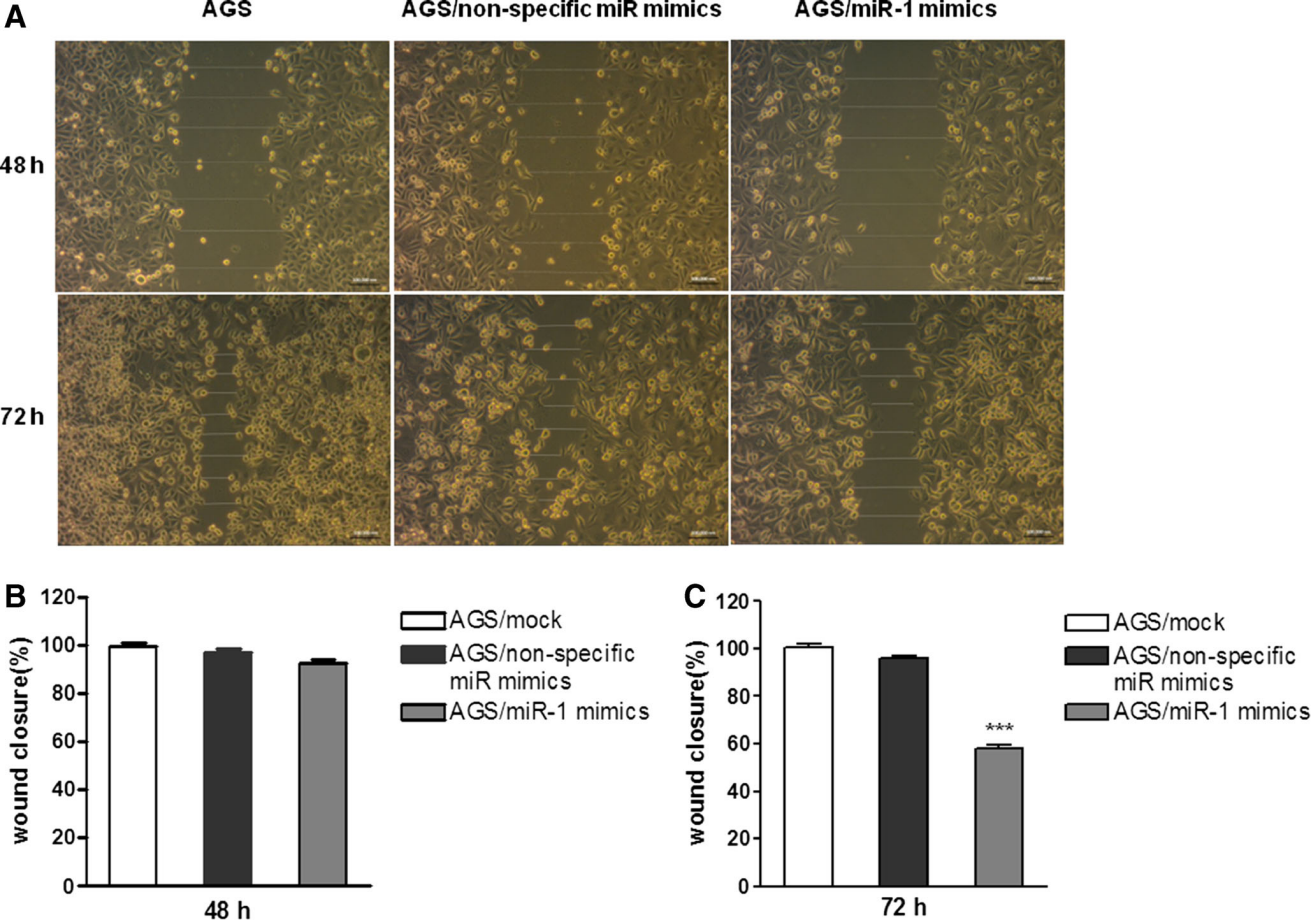
Ectopic expression of miR-1 reduces cell motility in the wound scratch assay. **a** AGS cells were seeded in six-well plates at 2 × 105 cells per well. Wounds were generated using a micropipette tip upon cell adherence. Then, cells were transfected with miR-1 and non-specific miR mimics. The extent of wound healing was monitored by phase contrast microscopy, and photomicrographs were acquired at 48 and 72 h. **b**, **c** Quantification of cell migration using the monolayer wound-healing assay. The data were then analyzed using Prism 5.0 software and expressed as mean ± SEM

To understand the potential function of miR-1 in gastric cancer, MTT assay was carried out to assess the proliferation of gastric cancer cells after transfection with miR-1 mimics. As shown in Fig. 1a, b, the expression levels of miR-1 were upregulated in these two gastric cancer cell lines as confirmed by SYBR Green real-time RT-PCR. Then we performed MTT to determine the function of miR-1. The results showed that the two gastric cancer cell lines were significantly inhibited in miR-1 transfectants in comparison with cells transfected with the non-specific miR negative control (Fig. 1c, d). These results indicated that overexpression of miR-1 inhibited proliferation of gastric cancer cells in a dose-dependent manner in vitro (Fig. 1). Furthermore, wound-healing assay data showed significantly different widths for the residual gaps obtained in the miR-1 transfection and non-specific miR control groups, especially at 72 h (Fig. 2, *P* < 0.001). This experiment was repeated for three times.

### MET is a direct target for miR-1 in gastric cancer

**Fig. 3:**
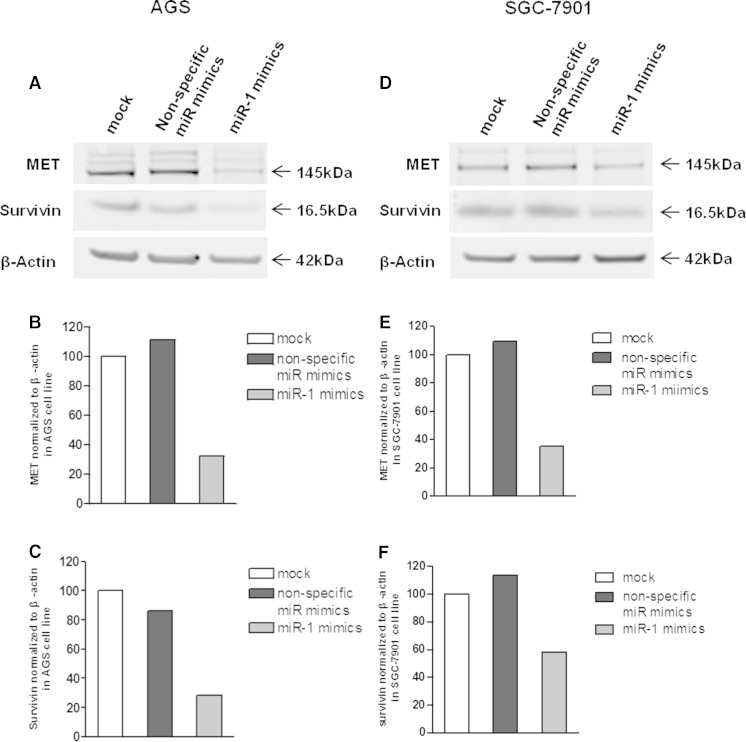
MET protein expression in miR-1 transfectants. **a** MiR-1 and non-specific miR mimics were transfected into AGS cells, respectively. MET and survivin protein expression levels were determined after 72 h. **b**, **c** Quantification of protein expression using the Odyssey Infrared Imaging System and the application software. The data indicate that MET and survivin protein expression levels were suppressed by 68 and 71.9 %, respectively. **d**, **e**, **f** MET and survivin protein expression in SGC-7901 cells. Both proteins were downregulated by 64.6 and 41.6 %, respectively

To assess the mechanism by which miR-1 inhibits gastric cancer cell growth, miR-1 mimics (40 nmol/L) were introduced into proliferating AGS and SGC-7901 cells. As shown in Fig. 3, MET protein expression was significantly downregulated in miR-1 transfectants in comparison with the control cells. These results indicated that MET is a common target gene for miR-1. In AGS and SGC-7901, the expression levels of MET were downregulated to 32 and 35.4 %, respectively.

### MET silencing inhibits gastric cancer cell growth

**Fig. 4:**
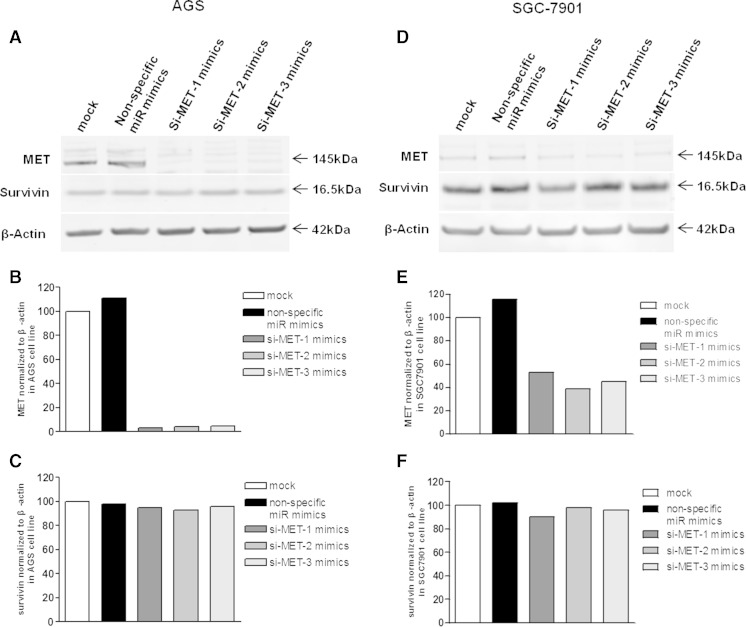
The MET protein was downregulated by siRNA in AGS and SGC-7901 cells. **a** si-MET-1/2/3 mimics and non-specific siRNA mimics were transfected into AGS cells. MET and survivin protein expression levels were determined after 72 h. **b** The data showed that MET protein expression levels were suppressed by 96.74, 95.86, and 94.95 %, respectively, for si-MET-1, 2, and 3. **c** Relative expression of survivin protein in AGS cells. **d** MET protein expression in SGC-7901 cells. **e** The protein expression was inhibited by 46.9, 61.2, and 54.8 %, respectively, after transfection with si-MET-1, 2, and 3. **f** Relative of survivin protein in SGC-7901 cells. The densities of the bands of MET and survivin expression were then quantified by Odyssey software 3.0 (LI-COR Biosciences). The data were then analyzed using Prism 5.0 software and expressed as mean ± SEM

**Fig. 5:**
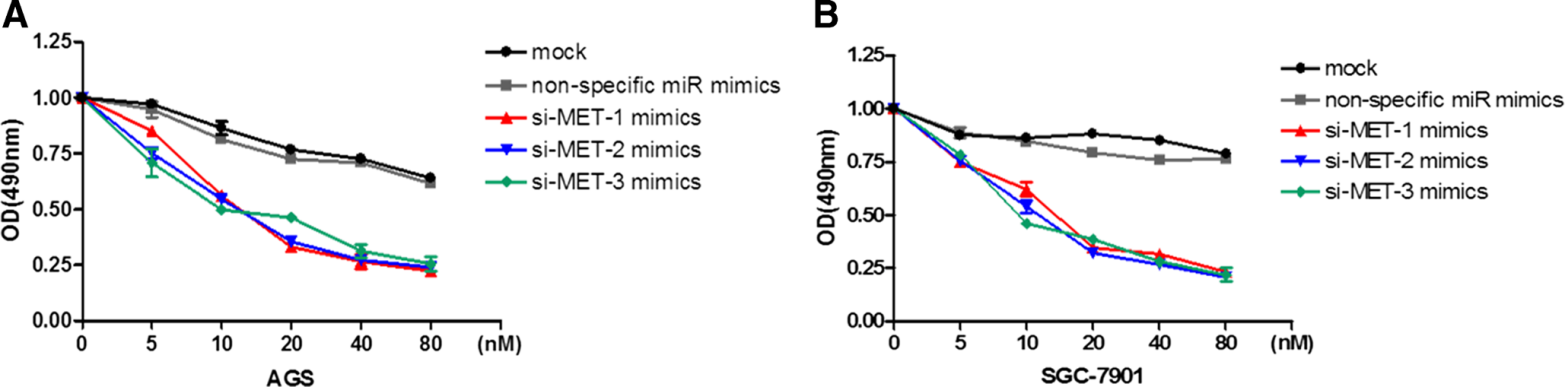
MET siRNA inhibits AGS and SGC-7901 cell growth. **a**, **c** AGS cells were transfected with si-MET-1/2/3 mimics and non-specific siRNA mimics at 5–80 nM, respectively, and incubated for up to 72 h in a medium containing 10 % FBS. **b** SGC-7901 cells were transfected with si-MET-1/2/3 mimics and non-specific miR mimics at 5–80 nM and incubated for 72 h. Cell growth was measured as described above

MET is known to be associated with carcinoma in various cancer types. The expression level of the MET protein was significantly decreased in both gastric cancer cell lines (AGS and SGC-7901) after transfection with miR-1 mimics (Fig. 3). In addition, the expression levels of survivin were also inhibited in both two cell lines (Fig. 3c, f). Conversely, MET expression was markedly reduced in MET siRNA (si-MET) transfectants. Specifically, inhibition of MET expression in AGS cells was more robust compared with that obtained for the SGC-7901 cell line (Fig. 4). AS expected, there are no significant changes on the expression levels of survivin in MET siRNA transfected cells. Loss-of-function assays using siRNA analysis were performed to examine the effect of MET on gastric cancer cell growth. The MTT assay revealed significant cell growth inhibition in si-MET-1/2/3 transfected cells after a 72-h transfection (Fig. 5).

### MET expression level is higher in gastric cancer tissues than matched specimens

**Fig. 6:**
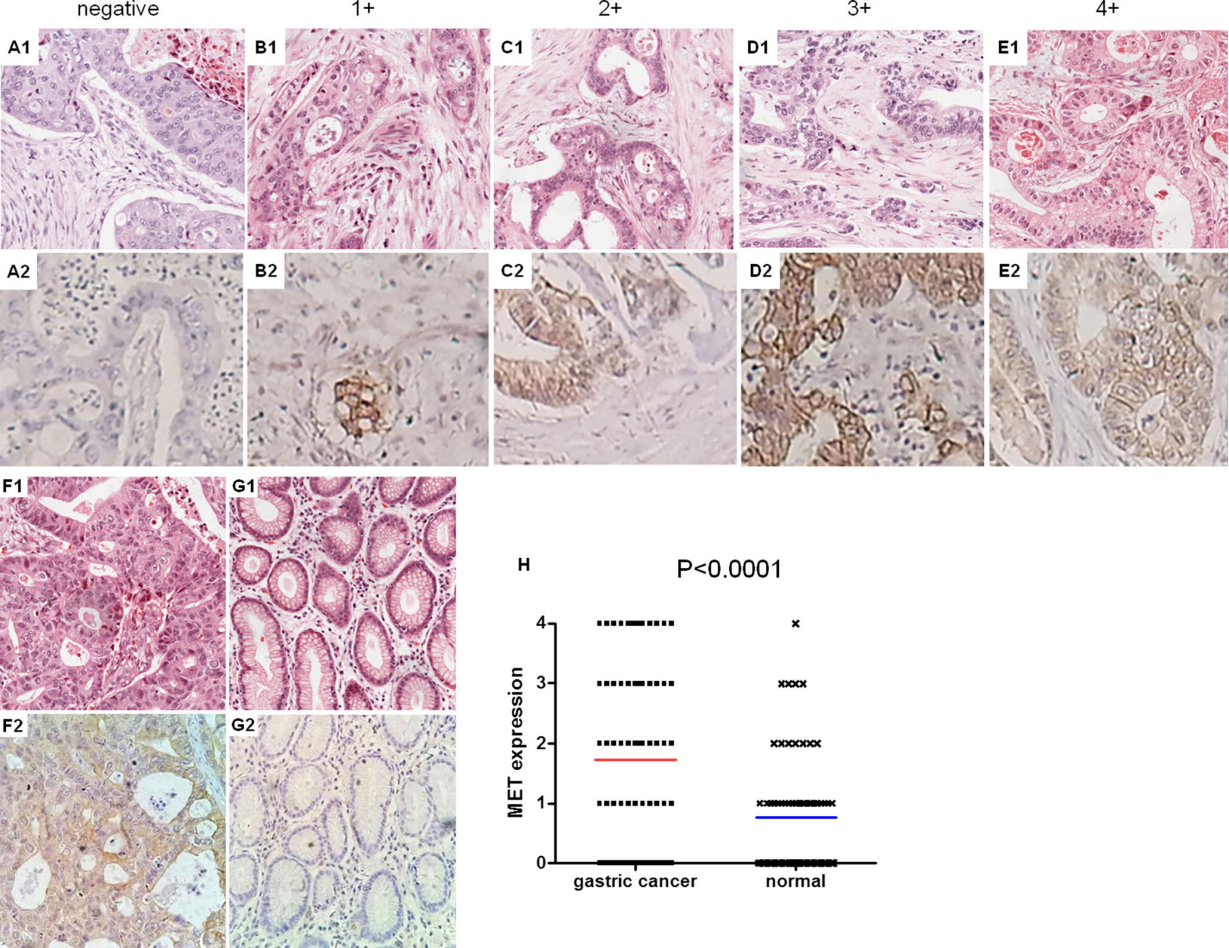
Expression of MET in gastric cancer tissues and adjacent specimens as analyzed by IHC. Representative results of MET staining micrographs of gastric tissues (×20). *A1*–*E1* Hematoxylin and eosin (*HE*) staining of gastric cancer tissues. *A2*–*E2* According to their staining, cells were divided into negative, 1+ (<25 % positive tumor cells), 2+ (25–50 % positive tumor cells), 3+ (50–75 % positive tumor cells), and 4+ (>75 % positive tumor cells). *F* and *G* represent gastric cancer tissue and matched adjacent samples. **h** It shows that average expression of MET in gastric cancer tissues were higher than the matched tissues (*P* < 0.0001)

We further investigated the underlying molecular mechanism of growth inhibition in gastric cancer cells by miR-1. MET is a well-known direct target gene for miR-1. We measured MET protein expression in the gastric cancer cell lines AGS and SGC-7901 by Western blot. We found that MET was significantly downregulated in miR-1 transfected cell lines (Fig. 3). We further determined MET expression in the tissue microarray (TMA) which consisted of 75 gastric cancer tissues with 75 matched adjacent non-gastric cancer tissues. The degree of immunostaining of TMA samples was evaluated separately by two independent investigators, who were blinded to patient details and sample’s histopathological features (Fig. 5). By comparing MET expression between gastric cancer tissues and adjacent non-gastric cancer specimens, we found that the average expression level of MET was higher in gastric cancer tissues (Fig. 6f, g).

## Discussion

Recent studies have demonstrated that different miRs contribute to many fundamental biological processes, including the carcinogenesis of gastric cancer [10, 18, 19]. In the present study, we found that miR-1 inhibits gastric cell proliferation and migration by targeting MET, in agreement with several recent studies suggesting that MET is a direct miR-1 target gene [16, 27].

Recent studies have shown the low expression level of miR-1 in other types of cancers compared with matched normal tissues [16, 28–30]. We also demonstrated that MET is highly expressed in gastric tumor tissues compared with matched normal tissues. Ectopic re-expression of miR-1 has been found to inhibit various types of cancers [31, 32]. Furthermore, multiple studies have shown that overexpression of miR-1 in non-miR-1-expressing lung cancer cells reverses their tumorigenic properties of growth, replication potential, motility/migration, clonogenic survival, and tumor formation in nude mice [30]. In hepatocellular carcinoma cells, miR-1 inhibits cell growth and reduces the replication potential and clonogenic survival [9, 29, 33]. Similarly, overexpression of miR-1 was shown to inhibit prostate cancer cell proliferation, migration, wound healing, and invasion activity [34]. Besides, ectopic expression of miR-1 has equal features of candidate tumor suppressor in other human cancers [32, 35]. Our current collectively showed that ectopic expression of miR-1 inhibited proliferation and migration in gastric cancer cells. miR-1 directly targets the MET gene and downregulates its expression.

MiRs cannot directly play their biological roles: they bind to the 3′-untranslated regions (UTRs) of target genes and inhibit gene expression by degrading the target mRNA or repressing its translation. It is therefore important to identify novel miR-mediated cancer pathways. In previous studies, miR-1 has been shown not only to target PIK3CA and inhibit the tumorigenic properties of lung cancer cells but also to be useful in predicting lymph node metastasis and postoperative recurrence in patients with NSCLC [36, 37]. In addition, miR-1 also targets TAGLN2 in head and neck squamous cell carcinoma (HNSCC) [35]. However, to our knowledge, the target gene of miR-1 in human gastric cancer has not been previously described. MET was significantly downregulated by ectopic expression of miR-1 in gastric cancer cell lines as shown above (Fig. 2).

MET, also known as hepatocyte growth factor receptor (HGFR), is a receptor tyrosine kinase (RTK) that is overexpressed and/or mutated in a variety of malignancies, including gastric cancer [38–40]. Expression of MET has been shown to be correlated with lymph node metastasis, distant metastasis, and cancer patients’ prognosis [40]. As shown above, the expression levels of the MET oncogene in gastric cancer tissues were higher than in matched tissues (Fig. 6h), indicating that overexpression of MET is related to gastric tumorigenesis. However, MET expression was not significantly different among various pathological grades. This might be due to the relatively limited samples number.

In conclusion, we showed that restoration of miR-1 expression in gastric cancer cells results in the inhibition of cell proliferation and migration. These findings support miR-1 as a tumor suppressor in gastric cancer. In addition, we demonstrated that MET may have an oncogenic function, which is directly regulated by miR-1. The identification of novel miR-1-regulated MET cancer pathways provides new insights into potential molecular mechanisms, target therapy, and prevention of gastric cancer.

## Abbreviations

miR: MicroRNA
mRNA: Message RNA
miR-1: miRNA-1
UTR: Untranslated region
